# Diversity and antimicrobial activities of culturable actinomycetes from *Odontotermes formosanus* (Blattaria: Termitidae)

**DOI:** 10.1186/s12866-022-02501-5

**Published:** 2022-03-25

**Authors:** Yanhua Long, Yong Zhang, Fang Huang, Song Liu, Tian Gao, Yinglao Zhang

**Affiliations:** 1 School of Life Sciences, Anhui Agricultural University, Hefei, 230036 China; 2 State Key Laboratory of Tea Plant Biology and Utilization, Anhui Agricultural University, Hefei, 230036 China

**Keywords:** Body surface, Termite's body surface, Diversity, Medium-based, *Streptomyces*, Pathogenic fungi, Active substances

## Abstract

**Background:**

Actinomycetes are important microbes, and they are very important for developing active substances for useful drugs. Actinomycetes are numerous inhabitants, and they are widely distributed in the nest of fungus-growing termites. Previously, we isolated and purified numerous actinomycetes from the combs of *Odontotermes formosanus* and obtained a variety of valuable natural products.

**Results:**

Here, we isolated and purified actinomycetes from fungus-growing termite *Odontotermes formosanus* using medium-based cultures. Among the eight media tested, M7 and I-HV media were found suitable for isolating actinomycetes*.* Further, 84 actinomycetes, including 79 *Streptomyces* isolates, were isolated and purified from *O*. *formosanus* and its combs, which belong to four genera (*Streptomyces*, *Kribbella*, *Amycolatopsis,* and *Cellulosimicrobium*). Then*,* the type and quantity of actinomycetes were positively correlated with the activity range of termites. Twenty-two actinomycetes strains showed antimicrobial activities. Among them, the BYF18, BYF48, BYF70, and BYF106 strains exhibited antifungal activities against five pathogenic fungi, with zone of inhibition (ZOI) values ranging from 3 to 21 mm. Grincamycin N was isolated and purified from the metabolites of *Streptomyces lannensis* (BYF106), and it displayed antibacterial activities against *Staphylococcus aureus* (ZOI = 13.82 ± 0.52 mm) and *Micrococcus tetragenus* (ZOI = 17.6 ± 0.5 mm) (gentamycin sulfate, as the positive control, had ZOI values of 19.9 ± 0.5 mm and 30.83 ± 0.75 mm, against *S. aureus* and *M*. *tetragenus*, respectively).

**Conclusions:**

Our results confirmed that the actinomycetes associated with *O. formosanus* are important sources of new active substances.

**Supplementary Information:**

The online version contains supplementary material available at 10.1186/s12866-022-02501-5.

## Background

Microorganisms are important sources of natural products, and they have great potential for application in medical, chemical, and agricultural industries [1]. For example, actinomycetes produce a wide variety of valuable natural products, including alkaloids, flavonoids, quinones, and peptides, which show diverse biological activities [2, 3]. Many antibiotics and pesticide ingredients are derived from actinomycetes [4, 5]. However, as more natural products are isolated and extracted from microorganisms inhabiting the aquatic, soil, and other common environments, there is a decrease in the probability of discovering new species and compounds.

Insects are by far the most diverse and abundant animal clade on earth, in terms of both the number of species and biomass [6]. They are tenacious and coexist with various microorganisms [7]. The microorganisms associated with insects are also diverse because of the complexity and diversity of their habitats and diets of their insect partners [8]. Thus, there is still a possibility of identifying new species and isolating new natural products from insect-related microorganisms [9]. Insect-related actinomycetes are important microbes and are very important for the development of active substances for new drugs [10, 11].

The fungus-growing termite *Odontotermes formosanus* (Blattaria: Termitidae) is abundantly found and is widely distributed in Asia and Africa [12]. It is a typical social insect with different grades, such as king, queen, workers, and soldiers, in its communities. All termites live in the stable environment of the nest, where there is a clear social division of labour. The king and queen are responsible for reproduction, soldiers are responsible for protecting the nest, and workers are responsible for collecting food, building the nest, and cultivating combs [13]. Actinomycetes are potential defensive symbionts of fungus-growing termites, because they produce antibiotic substances and are known to play the role of defensive microbes in other insect-fungus symbioses [14]. In our previous research, we isolated 23 strains of actinomycetes from the nests of *O*. *formosanus* and purified a compound (actinomycin D) with antifungal activity [10]. Here, we aim to assess the diversity and antimicrobial activity of actinomycetes isolated from body surfaces and guts of *O. formosanus* as well as the combs, through strain isolation, paired bioassay, chemical activity and identification.

## Results

### Isolation of actinomycetes strains

**Fig. 1:**
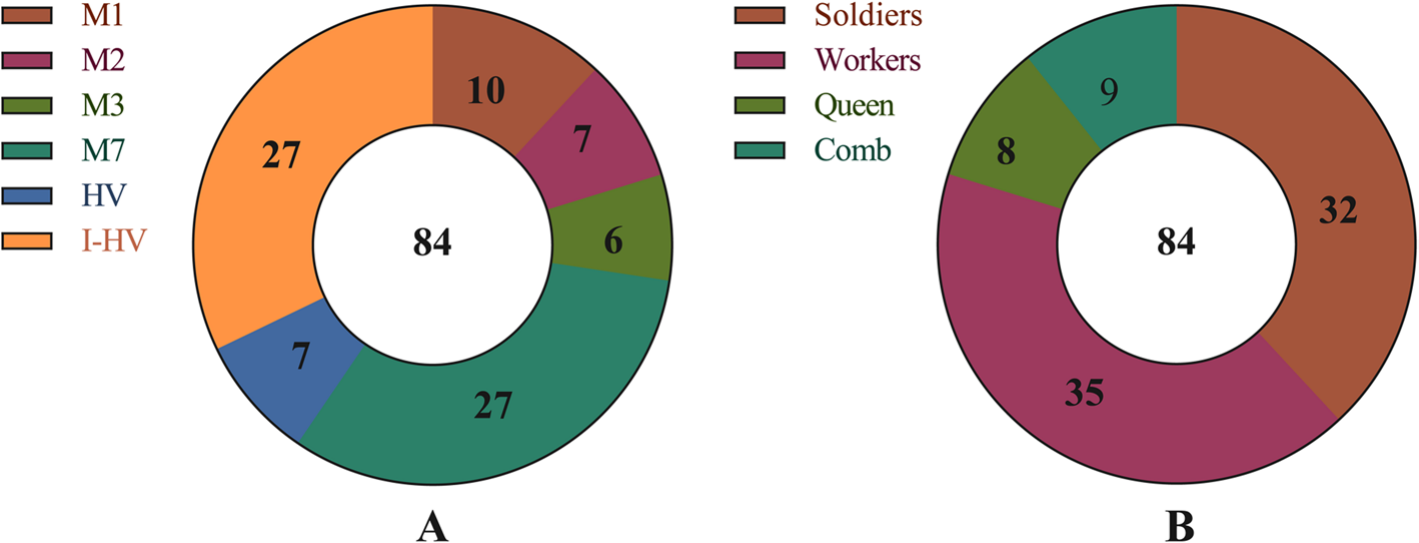
Actinomycetes were isolated from *O. formosanus* and combs. **A** Different isolation media. **B** Different isolation parts. Note: (i) No actinomycetes were isolated from ISP2 and ISP3 media; (ii) No actinomycetes were isolated from the king; (iii) No actinomycetes were isolated from the guts of *O. formosanus*, except those of the workers

Actinomycetes strains were isolated from body surfaces and guts of *O. formosanus* as well as the combs. According to the distinct morphological features of actinomycetes and colors of secondary metabolites, 84 actinomycetes strains, including 27, 27, 10, 7, 7, and 6 isolates from M7, I-HV, M1, M2, HV, and M3 media, were isolated. No strains were isolated from ISP2 and ISP3 media (Fig. 1A). Among these, 68 isolates were obtained from the termite’s body surfaces, including 32, 28, and 8 isolates from soldiers, workers, and queen, respectively. Further, 9 and 7 isolates were obtained from combs and guts of workers, respectively. No isolates were obtained from the king or guts of the *O. formosanus* soldiers, king, or queen (Fig. 1B).

### Identification and phylogenetic analysis of actinomycetes strains

**Fig. 2:**
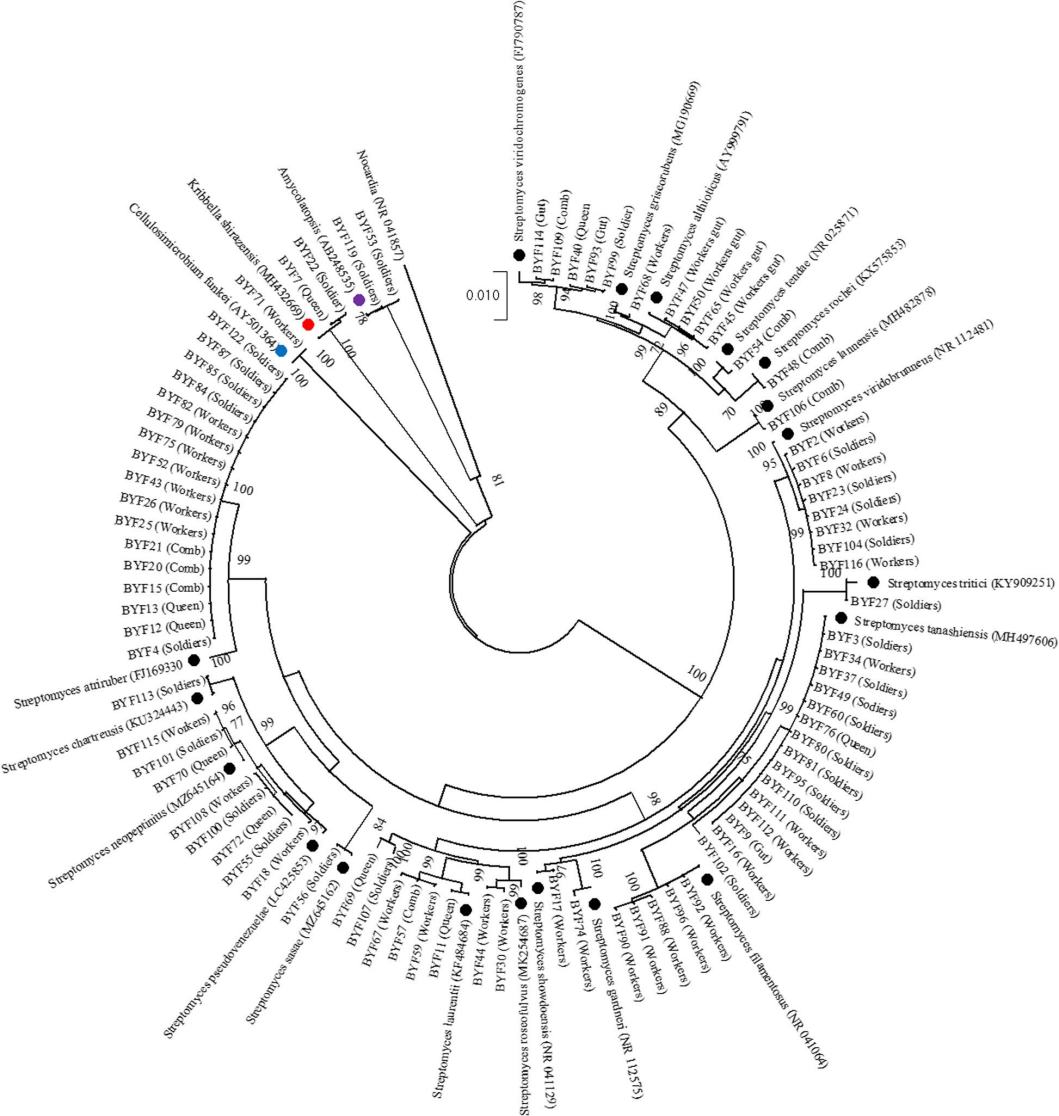
Maximum-likelihood tree based on the 16S rRNA sequences obtained from actinomycetes associated with *O. formosanus* and combs*.* The Tamura-Nei + G (T92 + G) model was used with 1,000 bootstrap replicates. Bootstrap values are shown at the nodes. Black: *Streptomyces*; Purple: *Amycolatopsis*; Blue: *Cellulosimicrobium*; Red: *Kribbella*

The 16S rRNA identification was amplified from 84 actinomycetes isolates. Of these isolates, 79 were identified as *Streptomyces*, which belong to 19 species, 2 were identified as *Kribbella shirazensis*, 2 were identified as *Amycolatopsis* sp*.,* and 1 was identified as *Cellulosimicrobium funkei* (Fig. 2). *Kribbella shirazensis was isolated from the* body surfaces of soldiers and queen. *Amycolatopsis* sp*.* was only isolated from the body surfaces of soldiers, and *Cellulosimicrobium funkei* was only isolated from the body surfaces of workers (Table S2).

**Fig. 3:**
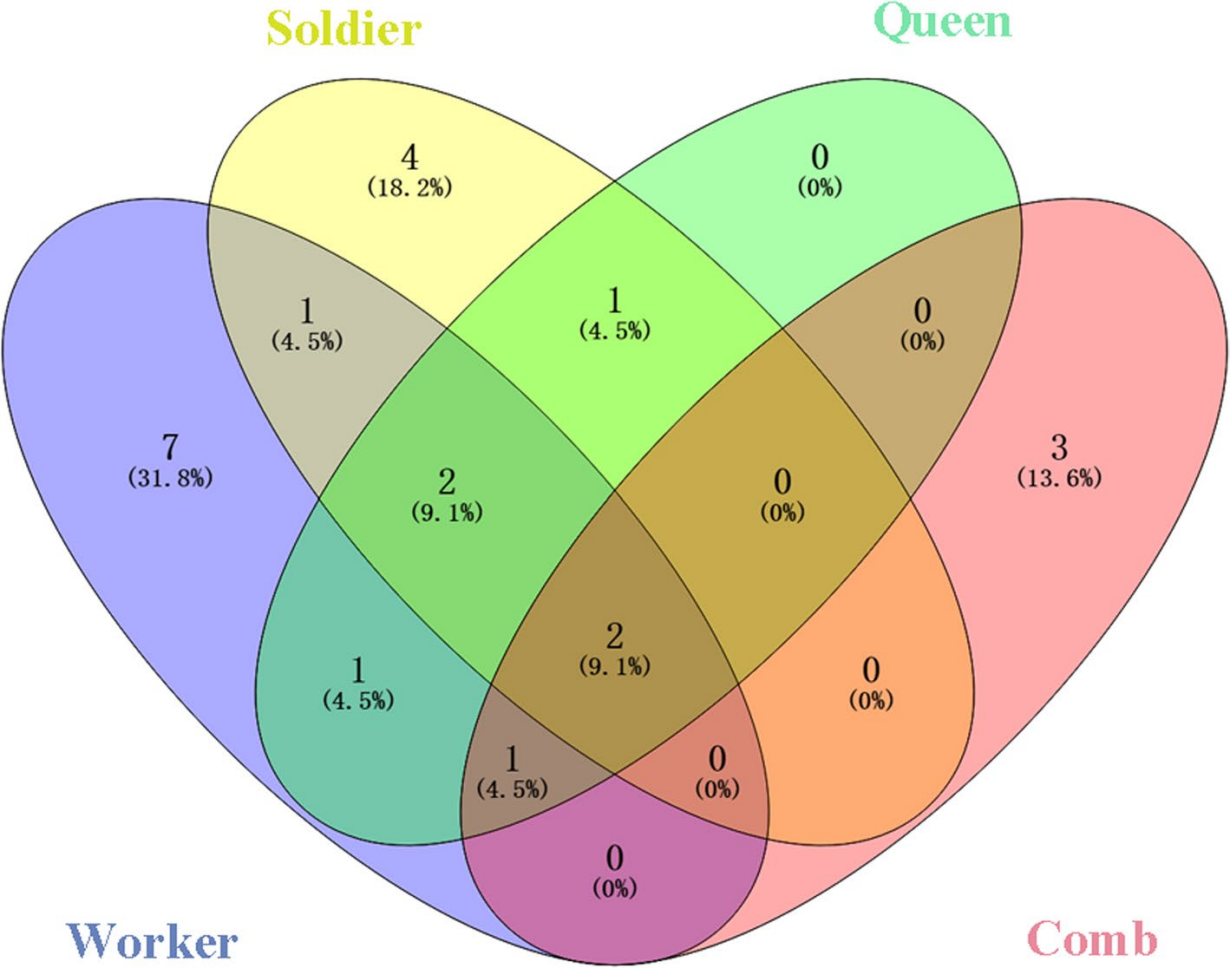
Venn diagram analysis of actinomycetes species isolated from *O*. *formosanus* and combs

A total of 75 actinomycetes isolates were obtained from *O. formosanus* and were identified as 19 different species, including 14, 10, and 7 species isolated from workers, soldiers, and queen, respectively. Moreover, 9 actinomycetes isolates were isolated from the comb and identified as 6 actinomycetes species (Fig. 3, Table S2).

### Exhibition of antifungal activity against pathogenic microbes by the isolated actinomycetes

To explore the antifungal activity of isolated strains, all isolates were challenged with various pathogenic microbes. Twenty-two *Streptomyces* isolates exhibited antifungal activity against pathogenic microbes.

Among these, 10, 6, 4, and 2 isolates were obtained from workers, soldiers, comb, and queen, respectively. Overall, these *Streptomyces* isolates showed strongest antifungal activity against *A. solani*, with zone of inhibition (ZOI) values greater than 20 mm (BYF74: ZOI = 21.9 ± 5.3 mm and BYF106: ZOI = 20.4 ± 0.5 mm), followed by *C. clavata* (BYF48: ZOI = 18.3 ± 1.6 mm), *C*. *lunata* (BYF70: ZOI = 16.3 ± 0.6 mm), *C*. *graminicola* (BYF48: ZOI = 12.0 ± 1.1 mm), and *F. oxysporum* f. sp. *cucumerinum* (BYF106: ZOI = 5.3 ± 0.6 mm) (Table 1).

**Table 1:** ZOI values of actinomycetes against growth of  plant pathogenic fungi (mm) (mean ± S.E.M., *n* = 3)

	**Strain**	***A. solani***	***F. oxysporum* f. sp. *cucumerinum***	***C. lunata***	***C. clavata***	***C. graminicola***
**Comb**	BYF20	13.0 ± 3.2 bc	NI^†^	NI	6.3 ± 0.6 cd	4.1 ± 0.1 e
	BYF48	18.5 ± 1.4 a	3.7 ± 0.6 ab	14.3 ± 1.9 a	18.3 ± 1.6 a	12.0 ± 1.0 a
	BYF54	16.8 ± 1.2 a	NI	NI	4.1 ± 0.1 d	NI
	BYF106	20.4 ± 0.5 a	5.3 ± 0.6 a	12.0 ± 3.5 b	11.1 ± 1.1 b	11.5 ± 1.1 a
**Queen**	BYF40	16.5 ± 0.5 a	NI	9.5 ± 2.4 c	7.7 ± 0.6 c	5.5 ± 0.5 d
	BYF70	9.1 ± 2.7 cd	4.7 ± 0.6 a	16.3 ± 0.6 a	16 ± 3.6 a	7.3 ± 2.0 c
**Workers**	BYF9	NI	NI	NI	NI	9.5 ± 0.5 b
	BYF17	13.0 ± 2.0 bc	NI	NI	NI	7.3 ± 0.6 c
	BYF18	9.3 ± 4.2 cd	4.1 ± 0.1 a	6.0 ± 0.2 d	15.7 ± 1.5 a	6.0 ± 1.7 d
	BYF 41	6.7 ± 0.6 e	NI	NI	NI	3.7 ± 0.6 e
	BYF44	NI	NI	NI	6.8 ± 0.3 cd	NI
	BYF59	NI	NI	NI	6.7 ± 1.2 c	5.7 ± 0.6 d
	BYF68	16.9 ± 3.1 a	NI	NI	NI	5.0 ± 1.0 d
	BYF71	NI	NI	NI	NI	8.2 ± 0.8 b
	BYF 74	21.9 ± 5.3	NI	NI	NI	NI
	BYF92	NI	NI	15.3 ± 2.2 a	NI	NI
**Soldiers**	BYF4	NI	NI	8.1 ± 0.2	NI	NI
	BYF23	NI	NI	6.5 ± 1.3 d	NI	9.6 ± 1.8 b
	BYF27	NI	2.3 ± 0.6 b	NI	4.0 ± 0.2 d	NI
	BYF31	13.5 ± 0.6 bc	NI	NI	NI	8.3 ± 1.2 b
	BYF113	13.0 ± 3.4 bc	4.7 ± 0.6 a	NI	9.7 ± 2.1 b	7.5 ± 0.5 c
	BYF119	11.1 ± 1.6 c	NI	NI	5.3 ± 0.6 d	NI

NI^†^: not inhibited

**Fig. 4:**
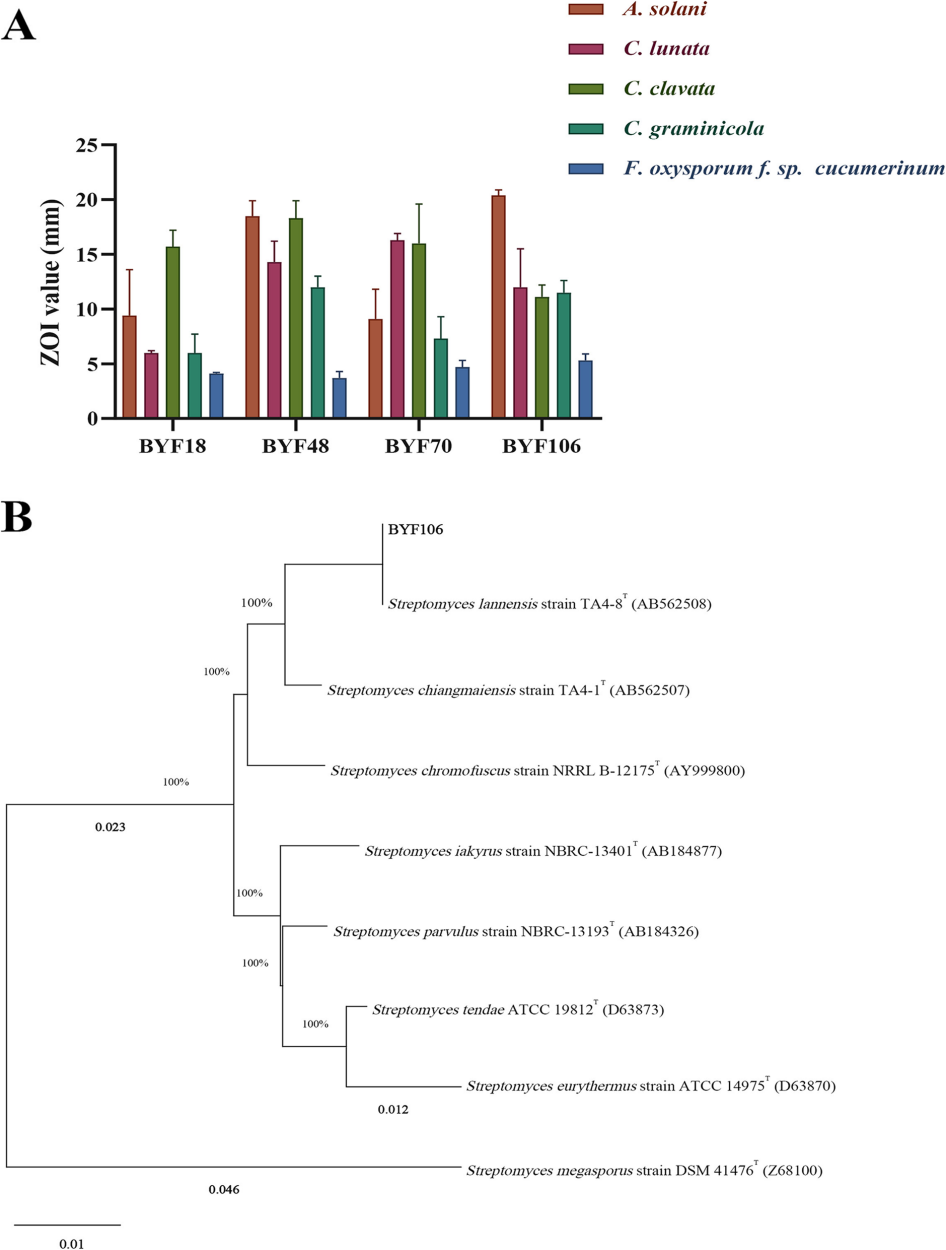
Antifungal activity of *Streptomyces* BYF18, BYF48, BYF70, and BYF106 against five  plant pathogens (**A**); Phylogenetic (ML) analysis of BYF106 (**B**). The Tamura-Nei + G (T92 + G) model was used with 1,000 bootstrap replicates. Bootstrap values are shown at the nodes. *Streptomyces megasporus* DSM 41476^ T^ was used an outgroup

Sixteen *Streptomyces* isolates showed inhibitory activity against two or more plant fungi (Table 1). Four *Streptomyces* isolates, BYF18, BYF48, BYF70, and BYF106, exhibited antifungal activity against all tested pathogenic fungi, with ZOI values ranging from 3 to 20 mm (Fig. 4A). Because the fermentation product yield of the BYF106 strain was higher than that of the BYF48 and BYF70 strains, we chose the BYF106 strain for an in-depth study. Meanwhile, according to the 16S rRNA gene sequence analysis, the BYF106 strain formed a cluster with *Streptomyces lannensis* type strain TA4-8. Thus, the BYF106 strain was *Streptomyces lannensis* (Fig. 4B).

### Exhibited antimicrobial activities of fermentation broth of BYF106

**Fig. 5:**
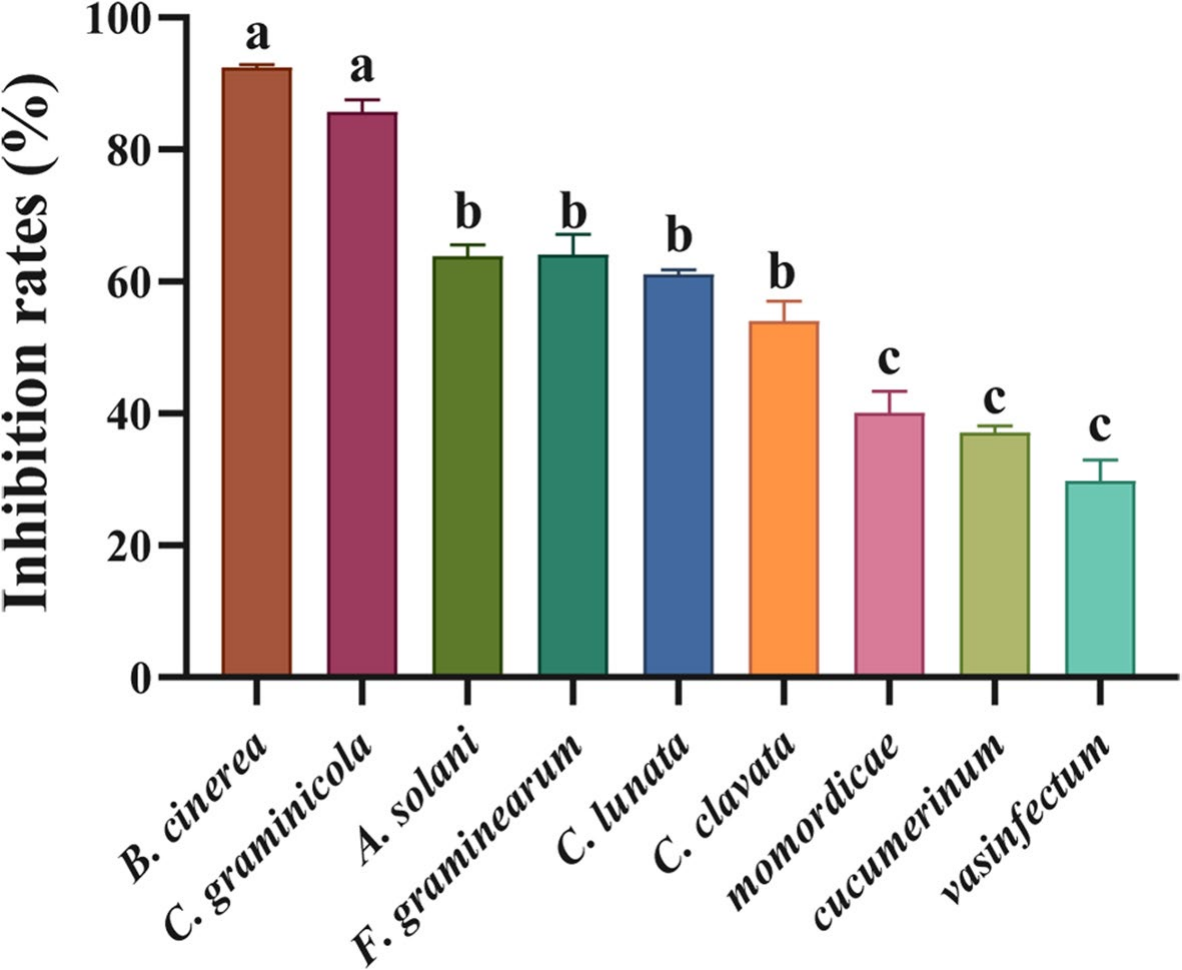
Inhibition rates (%) of the crude extract of BYF106 against growth of nine pathogenic fungi at 48 h of fermentation. Different letters indicate significant differences at *p* < 0.05. Note: *momordicae*, *cucumerinum*, and *vasinfectum* refer to *Fusarium oxysporum.* f. sp. *momordicae*, *Fusarium oxysporum.* f. sp. *Cucumerinum,* and *Fusarium oxysporum.* f. sp. *vasinfectum,* respectively

An extract from the fermentation broth of BYF106 showed antifungal activity against nine pathogenic fungi, with the highest inhibition rates at 48 h of fermentation (Table 2). Further, the inhibition rates of the BYF106 fermentation broth for *B. cinerea* and *C. graminicola* were up to 93% and 87%, respectively. It also showed good inhibitory activity against *A. solani*, *F. graminearum*, *C. lunata,* and *C. clavata,* with inhibition rates of more than 55%. Unfortunately, it had low antifungal activity against three *Fusarium oxysporum* species, with inhibition rates ranging from 29.81% ± 3.15% to 40.19% ± 3.12% (Fig. 5).

**Table 2:** Inhibition rates (%) of the crude extract of BYF106 against growth of nine pathogenic fungi (mean ± S.E.M., *n* = 3)

Pathogenic fungi	48 h	72 h	96 h
*B. cinerea*	92.46 ± 0.41%	81.09 ± 0.72%	59.58 ± 0.54%
*C. graminicola*	86.62 ± 0.32%	74.79 ± 1.05%	66.56 ± 0.64%
*A. solani*	64.26 ± 1.06%	48.87 ± 2.08%	48.53 ± 0.74%
*F. graminearum*	64.12 ± 3.03%	60.07 ± 0.91%	NI^†^
*C. lunata*	61.09 ± 0.74%	54.62 ± 0.92%	49.29 ± 0.81%
*C. clavata*	54.11 ± 3.01%	49.13 ± 2.02%	40.85 ± 1.01%
*F. oxysporum* f*.* sp*. momordicae*	40.19 ± 3.12%	38.37 ± 0.82%	29.71 ± 2.31%
*F. oxysporum* f. sp*. cucumerinum*	37.14 ± 1.08%	36.80 ± 4.03%	33.77 ± 2.01%
*F. oxysporum* f. sp*. vasinfectum*	29.81 ± 3.15%	18.19 ± 2.44%	18.98 ± 3.03%

NI^†^: not inhibited

### Structural elucidation of bioactive metabolites from BYF106

**Fig. 6:**
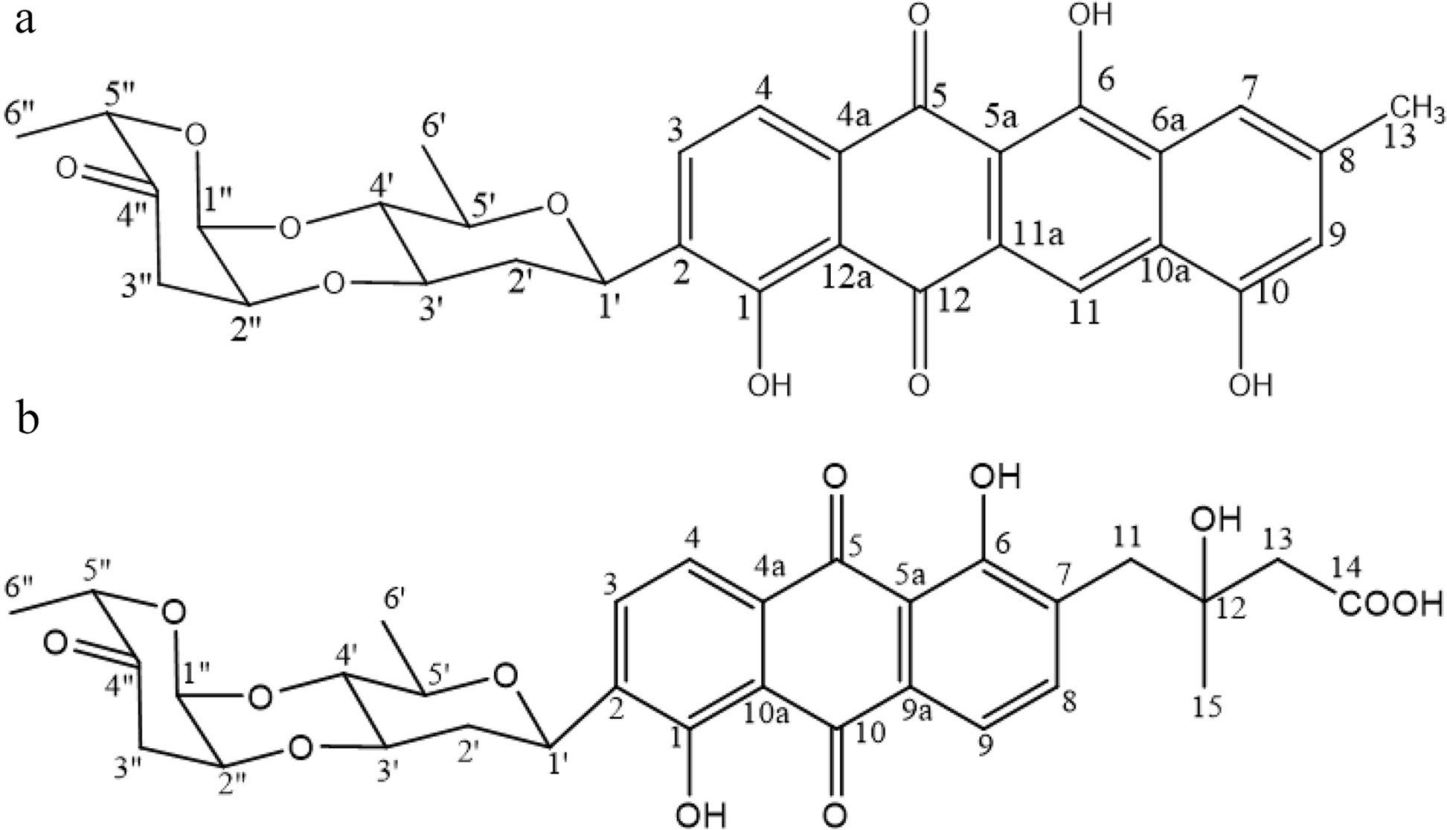
Compounds BYF106-1 and BYF106-2 isolated from the culture of actinomycetes BYF106. **a**: BYF106-1, grincamycin N (C_31_H_27_O_10_); **b**: BYF106-2, fridamycin D (C_31_H_32_O_12_)

Two compounds, BYF106-1 and BYF106-2, were isolated from the BYF106 fermentation broth. The chemical structures of BYF106-1 and BYF106-2  were shown in Fig. 6.

Compound BYF106-1 (grincamycin N) was obtained as a red powder, and its molecular formula, C_31_H_27_O_10,_ was deduced from an ESI–MS ion peak at *m*/*z* 559.1604[M-H]^−^. The ^1^H NMR and ^13^C NMR data were as follows: ^1^H NMR (600 MHz, DMSO-d6) δ1.25 (3H, d, J = 6.7 Hz, H-6″), 1.27(3H, d, J = 5.9 Hz, H-6’), 2.40 (3H, s, H-13), 3.51 (1H, t, J = 9.1 Hz, H-4’), 3.60 (2H, m, H-2’), 3.85 (1H, m, H-3’), 4.35 (1H, m, H-2″), 4.72 (1H, m, H-5″), 4.94 (1H, d, J = 11.2 Hz, H-1’), 5.23 (1H, d, J = 2.3 Hz, H-1″), 6.94 (1H, s, H-9), 7.50 (1H, s, H-7), 7.74 (1H, d, J = 7.9 Hz, H-4), 7.85 (1H, d, J = 7.9 Hz, H-3), 8.38 (1H, s, H-11), 10.88 (1H, s, HO-10), 13.40 (1H, s, HO-6); and ^13^C NMR (151 MHz, DMSO-d6) δ158.40 (C, C-1), 136.31 (C,C-2), 133.20 (CH, C-3), 118.33 (CH, C-4), 132.41 (C, C-4a), 186.21 (C, C-5), 108.71 (C,C-5a), 162.12 (C, C-6), 128.15 (C, C-6a), 114.22 (CH, C-7), 141.77 (C, C-8), 116.23 (CH, C-9), 155.89 (C, C-10), 124.10 (C, C-10a), 116.72 (CH, C-11), 125.02 (C, C-11a), 187.21 (C, C-12), 116.14 (C, C-12a), 21.85 (CH3, C-13), 70.55 (CH, C-1’), 35.84 (CH2, C-2’), 75.74 (CH, C-3’), 73.52 (CH, C-4’), 73.66 (CH, C-5’), 17.37 (CH3, C-6’), 90.46 (CH, C-1″), 70.86 (CH, C-2″), 40.05 (CH2, C-3″), 208.56 (C, C-4″), 76.91 (CH,C-5″), 16.05 (CH3, C-6″). These data were almost identical to those reported for grincamycin N (C_31_H_27_O_10_) [15].

Compound BYF106-2 (fridamycin D) was obtained as a yellow powder, and its molecular formula, C_31_H_32_O_12,_ was deduced from ESI–MS *m*/*z* 597.1971 [M-H]^+^. The ^1^H NMR and ^13^C NMR data were as follows: ^1^H NMR (600 MHz, DMSO-d6) δ1.19 (3H, s, H-15), 1.25 (3H, d, J = 6.7 Hz, H-6″), 1.27 (3H, d, J = 5.9 Hz, H-6’), 1.63 (1H, m, H-2’), 2.41 (2H, s, H-13), 2.47 (1H, t, J = 13.9 Hz, H-3’’), 2.51 (1H, m, H-2’), 2.91 (1H, t, J = 13.4 Hz, H-3″), 3.06 (1H, d, J = 13.1 Hz, H-11), 3.51 (1H, t, J = 9.2 Hz, H-4’), 3.86 (1H, m, H-3’), 4.34 (1H, m, H-2’’), 4.71 (1H, dd, J = 6.72 Hz, 6.73 Hz, H-5’’), 5.01 (1H, d, J = 10.9 Hz, H-1’), 5.22 (1H, d, J = 2.6 Hz, H-1’’), 7.74 (1H, t, J = 7.7 Hz, H-9), 7.82 (1H, t, J = 6.8 Hz, H-4), 7.92 (1H, s, H-3), 7.93 (1H, s, H-8); and ^13^C NMR (151 MHz, DMSO-d6) δ158.0 (C, C-1), 137.0 (C, C-2), 133.6 (CH, C-3), 118.8 (CH, C-4), 131.9 (C, C-4a), 187.8 (C, C-5), 115.0 (C, C-5a), 169.6 (C, C-6), 135.2 (C, C-7), 139.7 (CH, C-8), 118.3 (CH, C-9), 131.0 (C, C-9a), 187.8 (C, C-10), 115.4 (C, C-10a), 39.6 (CH_2_, C-11), 71.0 (C, C-12), 46.3 (CH_2_, C-13), 172.4 (COOH, C-14), 26.4 (CH_3_, C-15), 70.4 (CH, C-1’), 35.8 (CH_2_, C-2’), 75.7 (CH, C-3’), 73.5 (CH, C-4’), 23.6 (CH, C-5’), 17.3 (CH_3_, C-6’), 90.4 (CH, C-1″), 70.8 (CH, C-2″), 39.7 (CH_2_, C-3″), 208.7 (C, C-4″), 76.9 (CH, C-5″), 16.0 (CH_3_, C-6″). These data were almost identical to those reported for fridamycin D (C_31_H_32_O_12_) [16].

### Anti-bacterial activities of BYF106-1 and BYF106-2

**Fig. 7:**
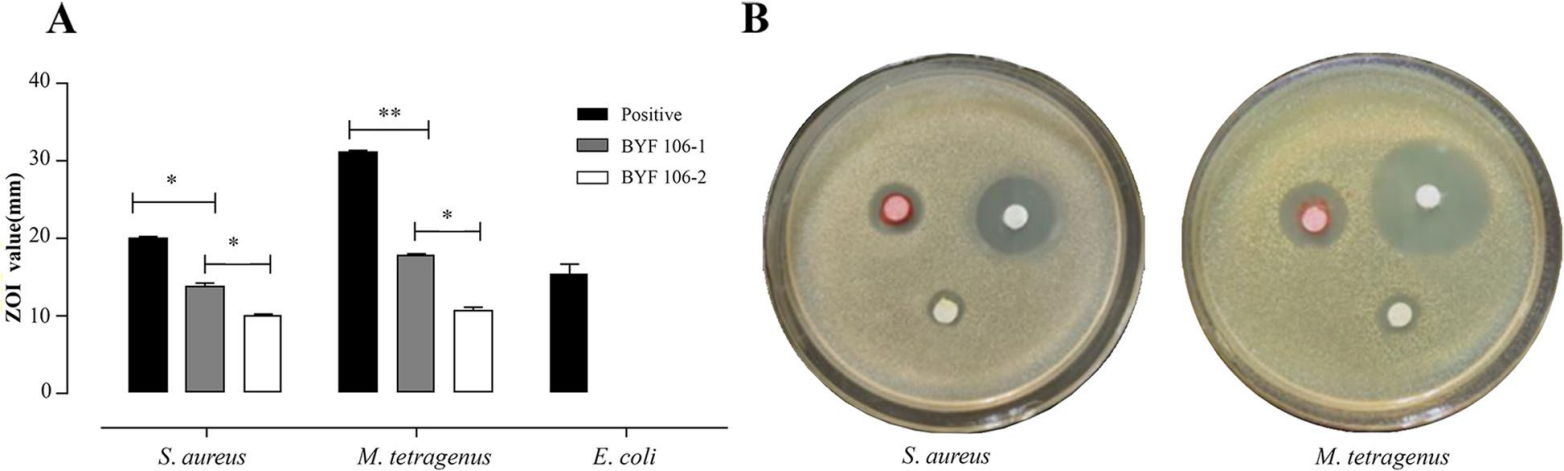
Zone of inhibition values of two compounds against two pathogenic bacteria (**A**, **B**). Values are the mean of three independent measurements ± S.E.M. *P < 0.05, **P < 0.01. Positive control was 30 µg gentamicin sulfate/filter paper (titer: USP grade, > 590 U/mg). **Red pill**: BYF106-1; **Yellow pill**: BYF106-2; **White pill**: gentamicin sulfate

Compounds BYF106-1 and BYF106-2 showed significant differences in their anti-bacterial activities against *S. aureus* and *M. tetragenus*. The ZOI value against *S. aureus* was significantly higher for BYF106-1 than for BYF106-2 (BYF106-1: 13.82 ± 0.52 mm vs. BYF106-2: 9.91 ± 0.31 mm, *P* < 0.05; gentamycin sulfate positive control: ZOI = 19.9 ± 0.5 mm). The ZOI value against *M. tetragenus* was also significantly higher for BYF106-1 than for BYF106-2 (BYF106-1: 17.6 ± 0.5 mm vs. BYF106-2: 10.7 ± 0.7 mm, *P* < 0.05; gentamycin sulfate positive control: ZOI = 30.83 ± 0.75 mm) (Fig. 7).

## Discussion

To obtain a wide range of actinomycetes, we used eight different media to isolate microbes from *O. formosanus* and fungus combs. We found that M7 and I-HV media were the most efficient for isolation of actinomycetes. We speculate that chitin in the M7 medium effectively inhibited the growth of some bacteria and fungi, while favoring the growth of actinomycetes, since they are known to fully absorb and utilize this substrate [17]. Starch is the only carbon source in the I-HV medium, and it favors mycelial extension and cell growth of actinomycetes. A medium containing amylase with starch as a delayed carbon source is suitable for bacteria that utilize starch, including oligotrophic actinomycetes [18]*.*

Pathogens are widely distributed in the soil. Soil-dwelling insects must overcome challenges arising from pathogens in the soil [19]. A key strategy of insects coping with environmental threats is the use of molecular defenses from symbiotic microbes, especially from insect-associated *Streptomyces* [20]. In this study, 84 actinomycetes strains were isolated from *O. formosanus* and its combs, and most of the isolates were *Streptomyces* spp*.* Significantly, majority of these isolates were isolated from the body surface of *O*. *formosanus*. This indicated that *O. formosanus*-associated actinomycetes are probably derived from the surrounding soil.

In the world of fungus-growing termites, all termites live in the stable environment of the nest, where there is a clear social division of labor. The king and queen are responsible for reproduction, soldiers protect the nest, and workers are responsible for collecting food, building the nest, and cultivating crops (*Termitomyces* genus) in fungus combs [13]. Hence, the workers and soldiers, which have a wide range of activities in the soil, are more vulnerable to pathogens. This can explain why majority of the isolates were obtained from workers (14 isolates) and soldiers (12 isolates). This can also explain why no actinomycetes were isolated from the king, which has a limited range of activities and a much smaller body than the queen.

Antifungal tests showed that many *Streptomyces* isolates, such as BYF106 and BYF74, exhibited positive antifungal activity, and they had ZOI values greater than 20 mm against *A. solani*. Four strains showed inhibitory activity against five plant pathogenic fungi (ZOI values ranging from 3 to 20 mm), which reflected their broad-spectrum antimicrobial activity. This result indicated that actinomycetes produce a variety of compounds, rather than a single compound, to antagonize specific pathogenic fungi. BYF106 and BYF48 strains also showed antifungal activity against four plant pathogenic fungi (ZOI values > 10 mm), indicating that actinomycetes associated with *O. formosanus* have great potential as sources of new active substances. The monomer compounds BYF106-1 (grincamycin N) and BYF106-2 (fridamycin D) produced by *S. lannensis* (BYF106) also showed inhibitory activity against two human pathogens. Thus, BYF106-1 also can be developed as an active substance.

## Conclusion

We found that culturable actinomycetes were mainly distributed on the body surfaces of *O. formosanus*, and the distribution of actinomycetes was affected by the termite’s role in the nest. Most of the isolated actinomycetes were *Streptomyces*, and many of them are known to have antimicrobial activity. Grincamycin N, a monomer compound with significant antibacterial activity, was isolated from the fermentation broth of BYF106. Our results highlight that actinomycetes associated with *O. formosanus* are good sources of new active compounds.

## Materials and methods

### Sample collection

The samples were collected in sterile petri dishes from the Jiangyin City, Jiangsu Province, China (31°55′N, 120°17′E).

Nine different phytopathogenic fungi, *Alternaria solani*, *Botrytis cinerea*, *Fusarium graminearum*, *Curvularia clavata*, *Curvularia lunata*, *Colletotrichum graminicola*, *Fusarium oxysporum* f. sp. *cucumerinum*, *Fusarium oxysporum* f. sp*. vasinfectum*, and *Fusarium oxysporum* f*.* sp*. momordicae*, and three pathogenic bacteria, *Escherichia coli*, *Staphylococcus aureus,* and *Micrococcus tetragenus*, were obtained from the Zhejiang Normal University, Jinhua City, Zhejiang Province, China (29°1′N, 119°6′E).

## Sample preparation

In total, 20 workers, 20 soldiers, 1 king, and 1 queen were removed from a freshly excavated termite nest and were separately placed in 10.0-mL sterile micro-centrifuge tubes for processing. Next, they were washed with sterile phosphate-buffered saline (PBS) buffer (pH 7.4) (consisting of 0.27 g KH_2_PO_4_, 1.42 g of Na_2_HPO4, 8 g of NaCl, and 0.2 g of KCl in 1L of distilled water) to obtain rinse solutions. Then, they were dissected under sterile conditions to obtain the whole guts, which were ground using sterile mortars to produce gut slurries. In addition, fungus combs (4 g) were ground and treated using four separate methods to obtain slurries. The following four methods were used: (i) 1 g of fungus comb was heated at 120 °C for 1 h before adding the 10 mL PBS buffer (pH 7.4); (ii) 1 g of fungus comb was subjected to microwave heating (120 W, 2,450 Hz) for 9 min before adding the 10 mL PBS buffer (pH 7.4); (iii), 1 g of fungus comb in 10 mL of 1.5% phenol was placed in a 30 °C water bath for 30 min; and (iv) 1 g of fungus comb in 10 mL of 0.05% SDS was placed in a 40 °C water bath for 20 min [21].

## Isolation and identification of actinomycetes

Actinomycetes were isolated by the serial dilution method using eight different media, M1, M2, M3, M7, HV, I-HV, ISP2, and ISP3, supplemented with 75 mg/L nystatin, 25 mg/L naphthyridinic acid, and 50 mg/L potassium dichromate (Table S1). The inoculated plates were incubated at 28 °C and were observed intermittently for the growth of actinomycetes.

Actinomycetes with distinct morphological features were selected for culture. Total genome DNA was extracted from samples using a Plant Genomic DNA kit (TIANGEN, China). All PCR reactions were carried out on an ABI Veriti thermocycler (USA) and using Phusion® High-Fidelity PCR Master Mix (New England Biolabs) and the specific primer pair 27F (5′-TCCTCCGCTTATTGATATGC-3′)-1492R (5′-GGTTACCTTGTTACGACTT-3′).

The 50-µL PCR reaction mixture contained 20.5 µL ddH_2_O, 25 µL Premix Taq (LA Taq Version 2.0), 2.5 µL DNA template, 1 µL 27F, and 1 µL 1492R. PCR was performed under the following conditions: 98 °C for 3 min, followed by 32 cycles of 98 °C for 10 s, 56 °C for 30 s, and 72 °C for 90 s, followed by 72 °C for 7 min and 16 °C for 4 min. The same volume of 1 × loading buffer (containing SYBR green) was mixed with PCR amplicons and electrophoresis was conducted on 1.2% agarose gels for amplicon detection. The products were sent to the General Biosystems Limited Company (Anhui, China) for sequencing, and the results were used for BLAST analysis and Ezbiocloud sequence alignment to construct the phylogenetic trees.

For phylogenetic reconstructions, the nucleotide substitution model determined by the Bayesian Information Criterion (BIC) value calculated by MEGA X software. The substitution model was Tamura 3-parameter and gamma distributed (G) (T92 + G) with 1,000 bootstrap replicates. The sequences of the 16 s rRNA gene of all test actinomycetes isolates were deposited in GenBank (accession number: MT498694-778).

## Screening for antifungal activities

The antifungal screening bioassay of actinomycetes isolates against phytopathogenic fungi was carried out by the disc diffusion method [22]. We used five different phytopathogenic fungi, *A. solani*, *C. clavata*, *C. lunata*, *C. graminicola,* and *Fusarium oxysporum* f. sp. *cucumerinum* as the test strains. The test pathogenic fungi were inoculated onto the centers of the potato dextrose agar (PDA) plate (Table S1) and then incubated in the dark at 28℃. Actinomycetes isolates were grown on Gause’s synthetic agar (Table S1) and then incubated in the dark at 28℃. When the soil-borne pathogenic fungi and actinomycetes were full of plates, a punch (6 mm) was used to obtain phytopathogenic fungi and actinomycetes cakes. Actinomycetes cakes were inoculated onto the edges were grown on the malt extract agar (MEA) medium (Table S1) and then incubated in the dark at 28℃. After 2 days of culture, the cakes of pathogenic fungi were inoculated onto the center of MEA media and then incubated in the dark at 28℃ for 3 days, and the diameter of the bacteriostatic circle with inhibitory activity was measured and recorded.

## Microbial fermentation of the BYF106 strain

Microbial fermentation of actinomycetes BYF106 was performed as described [23]. The slant culture of the BYF106 strain was inoculated in 150 mL Gause’s liquid medium in a 500 mL Erlenmeyer flask, with shaking at 180 rpm for 2 days at 28 °C. Afterwards, the inoculum was transferred as seeds into a 500 mL Erlenmeyer flask containing 150 mL of Gause’s liquid medium and grown at 28 °C at an agitation speed of 180 rpm for 10 days.

## Screening antimicrobial activities of BYF106 crude extracts

Crude pastes of the target strains (100 µg/mL) were screened for their antimicrobial activities. We used nine different phytopathogenic fungi, *A. solani*, *B*. *cinerea*, *F. graminearum*, *C. clavata*, *C. lunata*, *C. graminicola*, *F. oxysporum* f. sp. *cucumerinum*, *F*. *oxysporum* f. sp*. vasinfectum,* and *F*. *oxysporum* f*.* sp*. momordicae*.

The in vitro antifungal activities against phytopathogenic fungi were assayed using the growth rate method with slight modifications [24, 25]. Gentamycin sulfate was used as the positive control. The test pathogenic fungi were inoculated onto the centers of plates containing different media and then incubated in the dark at 28℃. When fungal mycelia reached the edges of control dishes, the antifungal activities were calculated [26]. The percentage of growth inhibition was calculated using the following formula:

Inhibition (%) = (1 − Da/Db) × 100.

where Da represents the diameter of the growth zone in the experimental dish (mm) and Db represents the diameter of the growth zone in the control dish (mm).

## Isolation and characterization of the active component of BYF106

The cultural broth (20 L) was extracted with ethyl acetate (4 × 20 L). Ethyl acetate extracts were combined and concentrated by evaporation of menstruum in vacuo. Then the crude extract (10 g) was chromatographed on a silica gel (200–300 mesh) column eluting with CH_2_Cl_2_/MeOH" mixtures with stably growing polarity (100:1—100:16, v/v) affording 16 parts (F1-F16). Further purification of part 2 (CH_2_Cl_2_/MeOH, 100:2, v/v)by silica gave pure compound 1 (9.7 mg), and further purification of part 4 (CH_2_Cl_2_/MeOH, 100:4, v/v)by silica gave pure compound 2 (5.6 mg).

Structural identification of the compound 1 and compound 2 was made by mass and nuclear magnetic resonance (NMR) spectroscopies (Agilent 600 MHz DD2, USA). The electrospray ionization mass spectrometry (ESI–MS) of the purified compound was recorded on a Mariner Mass 5304 instrument. ^1^H NMR and ^13^C NMR spectra were measured in CDCl_3_ with a Bruker AVANCE-400 (Burker, Switzerland) spectrometer, and the chemical shifts were obtained in δ (ppm) by referring to the solvent signal and tetramethylsilane (TMS) as internal standards [27].

## Antibacterial activities of the metabolite

Purified metabolites were screened for their antimicrobial activity. We used three common pathogenic bacteria (*E. coli*, *S. aureus* and *M. tetragenus*) as the test strains. The disc diffusion method was applied to evaluate the antimicrobial activities of isolated metabolites. Filter paper disks with metabolite dissolved in Dimethyl Sulfoxide (DMSO) at a concentration (30 µg/filter paper) were added to the Luria Broth (LB) (Table S1) medium, and the plates were incubated at 37ºC (*S. aureus* and *M. tetragenus*) for 24 h. Filter papers with DMSO, gentamycin sulfate was set as positive control. The zone of inhibition (ZOI) was determined by measuring the distance from the center of the disk to the end of the clear zone, and the experimental data were recorded using the “cross method” [28].

## Data analyses and statistics

All tests were performed in triplicate. Data are shown as mean values ± standard deviation.

Data were analyzed using SPSS 19.0, and figures were drawn using GraphPad Prism 9.0 and MEGA X.

## Supplementary Information

**Additional file 1:** **Table S1.** Medium formula used in the experiment.

**Additional file 2:** **Table S2.** List of all actinobacterial strains isolated from *Odontotermes formosanus* and combs.

## Data Availability

The datasets presented in this study can be found in online repositories. The names of the repository/repositories and accession number(s) can be found below: https:// www.ncbi.nlm.nih.gov/genbank/, MT498694-MT498778.
