# Supplementary material for: Diversity and antimicrobial activities of culturable actinomycetes from Odontotermes formosanus (Blattaria: Termitidae)
Source: BMC Microbiol. 2022 Mar 25;22:80. doi: 10.1186/s12866-022-02501-5 (PMC8951712; doi:10.1186/s12866-022-02501-5)
Supplement: Supplementary file 1 — Additional file 1: Table S1. Medium formula used in the experiment. [file 12866_2022_2501_MOESM1_ESM.docx]

**Supplementary Materials**

**Table S1** **Medium formula used in the experiment**

| **Medium (1 L)** | **Components** |
| --- | --- |
| **M1** | Cellulose 2.5 g, Sodium pyruvate 2.0 g, Asparagine 1.0 g, KNO_3_ 0.25 g, MgSO_4_•7H_2_O 0.2 g, K_2_HPO_4_ 0.2 g, CaCl_2_ 0.5 g, FeSO_4_•7H_2_O 0.01 g, Agar 15.0-20.0 g, H_2_O 1.0 L, pH 7.2 |
| **M2** | Cellulose 2.5 g, Sodium pyruvate 2.0 g, Arginine 1.0 g, KNO_3_ 0.25 g, MgSO_4_•7H_2_O 0.2 g, K_2_HPO_4_ 0.2 g, CaCl_2_ 0.5 g, FeSO_4_•7H_2_O 0.01g, Agar 15.0-20.0 g, H_2_O 1.0 L, pH 7.2 |
| **M3** | Citrate acid 0.12 g, Citric acid monohydrate 0.12 g, NaNO_3_ 1.5 g, K_2_HPO_4_•3H_2_O 0.4 g, MgSO_4_•7H_2_O 0.1 g, CaCl_3_•H_2_O 0.05 g, EDTA 0.02 g, Na_2_CO_3_ 0.2 g, Agar15.0-20.0 g, H_2_O 1.0 L, pH 7.2 |
| **M7** | Colloidal chitin 2.0 g, K_2_HPO_4_ 0.7 g, KH_2_PO_4_ 0.3 g, MgSO_4_•7H_2_O 0.5 g, FeSO_4_•7H_2_O 0.1 g, ZnSO_4_ 0.001 g, MnCl_2_ 0.001 g, Agar 15.0-20.0 g, H_2_O 1.0 L, pH 7.2 |
| **HV** | Humic acid 1.0 g (Dissolved in 10 mL of 0.2 NaOH), Na_2_HPO_4_ 0.5 g, KCl 1.71 g, MgSO_4_•7H_2_O 0.05 g, FeSO_4_•7H_2_O 0.01 g, CaCO_3_ 0.02 g, Agar 15.0-20.0 g, H_2_O 1.0 L, pH 7.2 |
| **I-HV** | Starch 2.0 g, KNO_3_ 0.5 g, KCl 1.7 g, MgSO_4_ 0.5 g, Na_2_HPO_4_ 0.5 g, CaCO_3_ 0.02 g, FeSO_4_ 0.01 g Multi-vitamins, Agar 15.0-20.0 g, H_2_O 1.0 L, pH 7.2 |
| **ISP2** | Yeast extract 4.0 g, Malt extract 10.0 g, Dextrose 4.0 g, Agar 15.0-20.0 g, pH 7.2 |
| **ISP3** | Oatmeal 20.0 g, Trace salt solution 1.0 mL, Agar 15.0-20.0 g, pH 7.2 (Trace salt solution: FeSO4•7H_2_O 0.1 g, MnCl_2_•4H_2_O 0.1 g, ZnSO_4_•7H_2_O 0.1 g, Distilled water 100 mL) |
| **Gause** | Soluble starch 20.0 g, KNO_3_ 1.0 g, K_2_HPO_4_ 0.5 g, MgSO_4_•7H_2_O 0.5 g, NaCl 0.5 g, FeSO_4_•7H_2_O 0.01 g, Agar 15.0-20.0 g, pH 7.4-7.6 |
| **PDA** | Potato 200 g, Glucose 20 g, Agar 15-20 g |
| **LB** | NaCl 10 g, Tryptone 10 g, Yeast extract 5 g, Agar 15-20 g |
| **MEA** | Raw malt 20 g, Sucrose 20 g, Peptone , Agar 15-20 g |
